# Development of an LC-MS/MS method for amantadine detection in human plasma using ZIF-8 as adsorbent and pharmacokinetic investigation

**DOI:** 10.3389/fphar.2026.1731595

**Published:** 2026-01-30

**Authors:** Zhengang Wang, Huanmei Han, Hongxia Li, Wenjuan Xu, Kuiying Ding

**Affiliations:** 1 Affiliated Hospital of Shandong Second Medical University, Weifang, Shandong, China; 2 Jinan International Travel Healthcare Center, Jinan, Shandong, China; 3 Weifang Customs, Weifang, Shandong, China

**Keywords:** amantadine, human plasma, LC-MS/MS, pharmacokinetics, therapeutic drug monitoring

## Abstract

In this study, a liquid chromatography-tandem mass spectrometry (LC-MS/MS) method was developed and validated for the quantitative determination of amantadine in human plasma, with the incorporation of an internal standard to improve analytical accuracy. Plasma samples collected from volunteers were processed using acetonitrile-methanol (3:1, v/v) as the extraction solvent, followed by protein precipitation and purification via the QuEChERS (Quick, Easy, Cheap, Efficacious, Rugged, and Safe) method. Analysis was performed using LC-MS/MS under multiple reaction monitoring mode, with a total run time of 8 min. Quantification was carried out using the internal standard method. After a single oral administration of 200 mg amantadine hydrochloride, plasma concentrations were measured at various time points. Pharmacokinetic parameters were derived by fitting the data to a pharmacokinetic model using specialized software. The results demonstrated good linearity over the range of 0.5–20 ng/mL, with a correlation coefficient (R^2^) of 0.9978. The extraction recovery ranged from 94.5% to 110.1%, and both intra-day and inter-day relative standard deviations (RSD) were below 10%. The limit of detection (LOD) and limit of quantification (LOQ) were 0.15 ng/mL and 0.5 ng/mL, respectively. The absorption and elimination processes of amantadine in plasma followed first-order kinetics, with R^2^ > 0.9. Notably, gender-specific differences were observed in the time to maximum concentration (T_max_) and maximum concentration (C_max_): females achieved a C_max_ of 670.23 ng/mL at 4 h, whereas males reached a C_max_ of 650.87 ng/mL at 8 h. This LC-MS/MS method is simple, rapid, and accurate, rendering it suitable for pharmacokinetic studies of amantadine in humans. Additionally, the established kinetic model provides valuable references for clinical medication guidance.

## 1 Introduction

**FIGURE 1:**
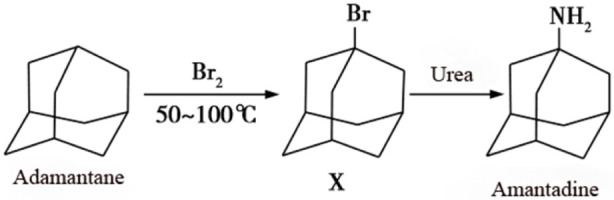
Synthesis of amantadine.

Amantadine, a broad-spectrum antiviral agent, is synthesized from adamantane via bromination and subsequent reaction with urea under high-temperature conditions (Figure 1). It exhibits potent antiviral activity against influenza A virus, RNA viruses (e.g., paramyxoviruses, coronaviruses, and flaviviruses), and DNA viruses (e.g., orthopoxviruses) (Zarmeena et al., 2024; Choi et al., 2025). This pharmaceutical agent can be administered via multiple routes for the treatment and prophylaxis of influenza A, alleviation of parkinsonian motor symptoms, and improvement of cognitive impairment following traumatic brain injury. In addition, it demonstrates efficacy against herpes zoster infections (Ian et al., 2025; Okhina et al., 2023; Gatto et al., 2024). However, high-dose or long-term administration of amantadine may induce serious adverse effects including myocardial infarction, mental confusion, and alopecia (Zhao et al., 2025). Plasma concentrations exceeding 1600 μg/L are deemed toxic (Wang et al., 2025). Consequently, reliable quantification of amantadine in blood is crucial for toxicity assessment, monitoring adherence, and evaluating therapeutic efficacy, which relies on maintaining efficacious plasma levels (Cesare et al., 2025). Thus, the development of an accurate, rapid, and robust analytical method for determining antiviral drug concentrations *in vivo* is of utmost importance.

The QuEChERS method has emerged as an efficient sample pretreatment technique, renowned for its minimal solvent consumption and reduced processing time (Gopi et al., 2024; Veena et al., 2023; Akbari et al., 2024). Initially introduced for pesticide residue analysis in agricultural products, it has subsequently been adapted for the determination of diverse analytes across various matrices. Yet, its application to biological samples for drug quantification remains relatively limited. Conventional dispersive solid-phase extraction (d-SPE) sorbents employed in QuEChERS demonstrate certain constraints, spurring research into novel high-performance purification materials (Su et al., 2024; Dai et al., 2025; Cai et al., 2024; Huo et al., 2025; Zhang et al., 2025).

**FIGURE 2:**
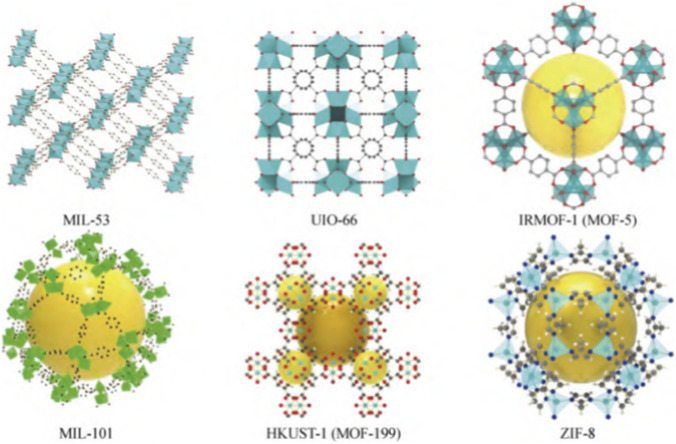
Schematic diagram of the 3D crystal structure of common MOFs.

Metal-organic frameworks (MOFs) represent a class of porous crystalline materials that are synthesized through the coordination of metal ions with multidentate organic ligands via either physical or chemical methods (Li et al., 2025). A typical crystal structure diagram of MOFs is presented in Figure 2. Their hierarchical porous structures endow MOFs with exceptionally high specific surface areas and outstanding extraction capacities, which, in turn, enhance the accessibility of active sites and molecular recognition capabilities (Zuo et al., 2025; Sun et al., 2025). These attributes underlie their remarkable adsorption performance toward biomacromolecules such as pigments and proteins, rendering them widely applicable in biomedical, food science, and photochemical fields (Swain et al., 2025; Hefayathullah et al., 2024). Zeolitic imidazolate frameworks (ZIFs), a subset of MOFs, are formed through the coordination of zinc ions with imidazole or its derivatives, featuring zeolite-like topological structures and substantial surface areas (Ying et al., 2025; Mao et al., 2023). Notably, ZIF synthesis typically avoids toxic solvents such as N,N-dimethylformamide (DMF) commonly used in conventional MOF preparation and can be rapidly synthesized under elevated temperatures (Jana et al., 2025; Obeso et al., 2025). Among these, ZIF-8 exhibits comparatively low toxicity and favorable biocompatibility, highlighting its potential in biomedical applications (Neuer et al., 2023; Wei et al., 2025; Zhao et al., 2024).

Plasma, as a complex matrix composed of proteins, carbohydrates, electrolytes, and other bioactive components, poses significant challenges for accurate drug quantification (Elbarbry et al., 2025; Zhu et al., 2024). Consequently, most existing methods for determining amantadine in plasma rely on solid-phase extraction (SPE) (Bhadoriya et al., 2018). But SPE features high costs, operational complexity, and substantial reagent consumption, all of which contribute to environmental pollution and hampers high-throughput processing. In this study, we introduced ZIF-8, a metal-organic framework with exceptional clean-up efficiency, as a QuEChERS adsorbent for sample pretreatment. Coupled with LC-MS/MS detection, we developed a robust, sensitive, environmentally friendly, and reproducible method for quantifying amantadine in human plasma. This validated method was successfully applied to a pharmacokinetic study of amantadine in healthy Chinese volunteers, facilitating the characterization of its pharmacokinetic profile and the establishment of a preliminary mathematical model to support precision dosing in clinical practice. Our pharmacokinetic findings in healthy volunteers, demonstrating biphasic behavior with rapid absorption and first-order elimination, are consistent with the established profile of amantadine. The observed gender difference in T_max_ was in line with known physiological variations affecting gastric emptying and absorption. Building upon and extending previous pharmacokinetic studies which primarily described the concentration-time curve, the present study integrated these empirical observations into a validated predictive mathematical model. This model serves as a translational bridge between descriptive pharmacokinetics and clinical application-a tool that was not emphasized in the earlier methodological studies cited. It enables a more dynamic assessment of patient exposure, potentially enhancing utility of single time-point measurements.”

## 2 Materials and methods

### 2.1 Materials and equipment

Chromatography-grade methanol, acetonitrile, and ethyl acetate were products of Fisher Scientific (Waltham, United States). Analytical-grade formic acid and glacial acetic acid were products of Tedia Company (Fairfield, United States). Analytical-grade anhydrous magnesium sulfate, purchased from Guangfu Fine Chemical Research Institute (Tianjin, China), was pretreated by drying in a muffle furnace at 500 °C for 5 h. Cleanert S-C18 adsorbents (50 μm, 60 Å) were obtained from Agela Technologies Inc. (Tianjin, China). ZIF-8 adsorbent (coprecipitation method, 100–400 nm) was purchased from XFNano (Nanjing, China). ProElut C18 reversed-phase silica-bonded solid-phase extraction (SPE) columns (particle size: 50 μm, pore size: 60 Å, specific surface area: 500 m^2^/g, 200 mg/6 mL) were acquired from Dikma Technologies Inc. (Beijing, China). Analytical-grade ammonium acetate was purchased from Rui Jinte Chemicals (Tianjin, China). Blank plasma samples were collected from healthy volunteers. Amantadine standard (>98% purity) and amantadine-D_15_ internal standard (98.2% purity) were products of Sigma-Aldrich (St. Louis, United States).

Equipment: Liquid chromatography-tandem mass spectrometry (LC-MS/MS, Agilent 1290-6,460, Shanghai, China) equipped with an electrospray ionization (ESI) source; XS205DU analytical balance (Mettler Toledo, Switzerland); 5810R refrigerated centrifuge (Eppendorf, Hamburg, Germany); N-EVAP nitrogen evaporator (Organomation Associates, Berlin, United States); Milli-Q water purification system (Millipore, Billerica, United States); KQ-500 ultrasonic cleaner (Kunshan Ultrasonic Instruments, Suzhou, China); Cleanert PCX-SPE cartridges (6mg/3mL, Bonna-Agela Technologies, Tianjin, China).

### 2.2 Instrumental conditions

#### 2.2.1 Chromatographic conditions

Chromatographic separation was performed on an Agilent 1260 HPLC system equipped with an Agilent ZORBAX SB-C18 column (100 mm × 2.1 mm, 3.5 μm). The column temperature was maintained at 40 °C, and the autosampler was set at 10 °C. The mobile phase consisted of (A) 5 mM ammonium acetate in water and (B) methanol. The gradient elution program was as follows: 0–3.0 min, 25% B; 3.0–3.01 min, 75% B; 3.01–5.0 min, 75% B; 5.0–5.01 min, 25% B; run time: 8 min. The flow rate was 0.3 mL/min, and the injection volume was 5 µL.

#### 2.2.2 Mass spectrometric conditions

Ionization source: Electrospray ionization (ESI), positive polarity; Scan mode: Multiple reaction monitoring (MRM); Curtain gas: 35 psi; Ion spray voltage: 5500 V; Collision gas: Nitrogen; Drying gas temperature: 550 °C; Nebulizer gas (GS1): 55 psi; Auxiliary gas (GS2): 55 psi. The MRM transitions and optimized parameters for amantadine are summarized in Table 1.

**TABLE 1:** Partial mass spectrum parameters of amantadine.

Compound	Precursor ion (m/z)	Product ion (m/z)	Retention time (min)	Declustering potential (DP, V)	Collision Energy (CE,V)
Amantadine	152.1	135.0^*^, 93.0	2.89	80	22, 35
Amantadine-D_15_(IS)	167.1	150.3	2.89	80	25

* Quantitative ion.

### 2.3 Experimental methods

#### 2.3.1 Preparation of solutions

##### 2.3.1.1 Preparation of stock solutions

###### 2.3.1.1.1 External standard stock solution (100 μg/mL)

Amantadine standard (0.01 g) was accurately weighed into a 100 mL volumetric flask, dissolved in a small volume of methanol, and brought to volume with methanol. The solution was mixed thoroughly to obtain a standard stock solution at a concentration of 100 μg/mL.

###### 2.3.1.1.2 Preparation of the isotopically labeled internal standard stock solution (10 μg/mL)

Amantadine-D_15_ internal standard (1 mg) was accurately weighed into a 100 mL volumetric flask, dissolved in a small volume of methanol, and subsequently brought to volume with methanol. The solution was mixed thoroughly to obtain an internal standard stock solution at a concentration of 10 μg/mL.

##### 2.3.1.2 Preparation of intermediate standard solutions

###### 2.3.1.2.1 External standard intermediate solution (1.0 μg/mL)

Using a pipette, 1.0 mL of the amantadine external standard stock solution (100 μg/mL) was accurately transferred into a 100 mL volumetric flask and brought to volume with methanol to prepare an external standard intermediate solution at a concentration of 1.0 μg/mL.

###### 2.3.1.2.2 Internal standard intermediate solution (1.0 μg/mL)

A volume of 1.0 mL of the amantadine-D_15_ stock solution (100 μg/mL) was accurately pipetted into a 100 mL volumetric flask and diluted to volume with methanol to obtain an internal standard intermediate solution at a concentration of 1.0 μg/mL.

##### 2.3.1.3 Preparation of matrix-matched calibration standards

Blank sample matrix was processed in accordance with the sample preparation procedure described in Section 2.3.2 Aliquots (1 mL each) of the resulting blank matrix extracts were transferred into separate vials and evaporated to near-dryness under a gentle stream of nitrogen. Equal volumes of the internal standard working solution, an appropriate volume of the amantadine standard intermediate solution (for generating the calibration series), and a mixture of ammonium acetate in water (containing 0.1% formic acid) and methanol (70:30, v/v) were added. The mixture was reconstituted and diluted to a final volume of 1 mL with the aforementioned ammonium acetate-methanol solution, followed by thorough vortex mixing to ensure homogeneity, producing the matrix-matched calibration standards. The calibration curve was generated by plotting the peak area ratio of the analyte to the internal standard (y-axis) versus the corresponding nominal concentration of the analyte (x-axis).

##### 2.3.1.4 Preparation of extraction and reconstitution solutions extraction solution

Acetonitrile (300 mL) and methanol (100 mL) were mixed thoroughly.

Reconstitution Solution: 25 mL of 0.1% formic acid aqueous solution and 75 mL of methanol were mixed thoroughly.

#### 2.3.2 Optimization of sample protein precipitation and extraction reagents

Plasma samples (1 mL) were aliquoted into 10 mL plastic centrifuge tubes. Subsequently, 2 mL of protein precipitation reagent was added to each plasma sample. The resulting supernatant was then transferred into a new 10 mL centrifuge tube. After that, 5 mL of extraction solvent was added to the supernatant. The mixture was vortexed for 2 min to ensure thorough mixing and centrifuged for 5 min at 4 °C using a high-speed refrigerated centrifuge. The supernatant was carefully collected for subsequent purification steps, while the solid pellet was discarded.

#### 2.3.3 Selection of sample purification conditions

##### 2.3.3.1 Solid-phase extraction (SPE) method

PCX cation-exchange solid-phase extraction cartridges were preconditioned sequentially with 3 mL of methanol and 3 mL of water. The entire supernatant obtained from Section 2.3.2.1 was then loaded onto the preconditioned cartridge. The cartridge was subsequently washed sequentially with 3 mL of 2% formic acid in water (v/v), 3 mL of methanol, and 3 mL of n-hexane at a flow rate of 1 mL/min. Finally, the target analytes were eluted with 5 mL of 5% ammonia in methanol (v/v). The eluate was evaporated to dryness under a gentle stream of nitrogen, and the residue was reconstituted in 1 mL of the reconstitution solution. After passing through a 0.22 μm membrane filter, the resulting working solution was collected for instrumental analysis.

##### 2.3.3.2 QuEChERS method

An accurate volume of the aforementioned extract was transferred into a 10 mL glass tube containing a mixture of 40 mg ZIF-8 (synthesized by coprecipitation method) and 10 mg C18. The tube was vortexed vigorously for 2 min and then allowed to stand for 5 min. Subsequently, supernatant was transferred to a new 10 mL glass centrifuge tube and evaporated to near-dryness under a stream of nitrogen. The residue was reconstituted in 1 mL of a mixture of 0.1% formic acid in water and methanol (the specific ratio should be clarified if known, e.g., 70:30, v/v; it should be specified; otherwise, it can be described generally). It was then vortexed for 1 min to ensure complete dissolution, and finally filtered through a 0.22 μm organic membrane filter prior to instrumental analysis.

#### 2.3.4 Matrix-matched calibration standards

Blank plasma was processed in accordance with Section 2.3.2.1. The processed blank plasma was spiked with the amantadine standard (1 μg/mL) and internal standard to prepare matrix-matched calibration curves covering the concentration range of 0.5–20.0 ng/mL. All standards were analyzed, and the peak area ratios (analyte/internal standard) were plotted against the corresponding concentrations to construct the calibration curve.

#### 2.3.5 Matrix effect evaluation

A series of working solutions with concentrations of 0.5, 1.0, 2.0, 5.0, 10.0, and 20.0 ng/mL were prepared using both blank matrix extract and reconstitution solution. Subsequently, the matrix-matched standard solutions (containing the target compound) and the reagent blank standard solutions were analyzed. The peak area ratios of the analyte to the internal standard were determined for each solution, and calibration curves were generated by plotting these ratios against the corresponding concentrations of the working solutions.

#### 2.3.6 Validation of method effectiveness

After optimizing the experimental conditions, an analytical method was established and applied for the determination of amantadine in plasma. A series of matrix-matched standard solutions of amantadine at concentrations of 0.5, 1, 2, 5, 10, and 20 ng/mL were prepared and analyzed. A calibration curve was constructed by plotting the average peak area ratio (Y) of the quantitative ion of amantadine to the ion of the internal standard against the corresponding mass concentration (X, ng/mL).

To determine the recovery of amantadine from plasma samples, the standard-addition method was used. The standard working solutions (for calibration standards and QC samples) and the internal standard working solution were spiked into blank plasma at a volume of 10 µL per 990 µL of plasma (i.e., a spiking ratio of 1:99, v/v) and amantadine standard solutions with four concentration levels of 0.5, 1.0, 2.0, and 5.0 ng/mL. Six replicates for each concentration were analyzed in 1 day, and the experiment was repeated at weekly intervals to evaluate inter-day precision.

#### 2.3.7 Stability studies

Assess amantadine stability in plasma under:

Short-term: 12–48 h at 22 °C and 2 °C–8 °C.

Long-term: 1–7 days at 2 °C–8 °C.

Freeze-thaw: Three cycles (−20 °C to room temperature).

Post-processing: 24 h at 2 °C–8 °C.

#### 2.3.8 Pharmacokinetic study

A single oral dose of 200 mg amantadine (an antiviral agent) was administered to 20 healthy volunteers aged 18–60 years. Plasma samples were collected at 0, 0.5, 1, 2, 4, 8, 12, 24, and 48 h post-administration. Each plasma sample was analyzed in triplicate, and the mean values were calculated. The data were subsequently stratified by gender for pharmacokinetic analysis and mathematical model establishment. All procedures involving human participants were reviewed and approved by the Ethics Committee of the Affiliated Hospital of Shandong Second Medical University in accordance with the ethical standards (No. KY-2024-M127); Written informed consent was obtained from all individual participants (or their legal guardians) prior to any study-related procedures. The consent form detailed the research purpose, experimental procedures, potential risks and benefits, confidentiality provisions, and the right to withdraw from the study at any time without prejudice.

## 3 Results and discussions

### 3.1 Optimization of the liquid phase section

The composition of the mobile phase, including buffer concentration and pH, plays a pivotal role in augmenting quantification specificity and optimizing mass spectrometry (MS) conditions, as it directly impacts the peak shape of analytes during chromatography and their ionization efficiency in MS. We systematically evaluated volatile ammonium salts, which are compatible with MS and facilitate the ionization of analytes. Unlike methods that solely employ acid modifiers (e.g., formic acid), the addition of 5 mM ammonium acetate was found to be particularly efficacious. This additive not only improved the peak shape by suppressing silanol interactions-a known issue for basic compounds on reversed-phase columns-but also provided a proton-rich environment in the gas phase, enhancing ionization stability. This resulted in a significant reduction in peak width and an improvement in peak symmetry, thereby increasing the precision of peak integration. Furthermore, in contrast to the widespread use of acetonitrile in high-throughput bioanalytical methods, methanol demonstrated superior elution performance under our gradient conditions, enabling better separation from potential endogenous interferences in plasma. Consequently, a mobile phase consisting of methanol: 5 mM ammonium formate (25:75, v/v) was selected. This composition balances the excellent peak-focusing capability of the ammonium buffer and the strong eluting power of methanol, ensuring consistent sensitivity and efficacious retention of amantadine while maintaining system backpressure within an acceptable range.

### 3.2 Optimization of mass spectrometric conditions

**FIGURE 3:**
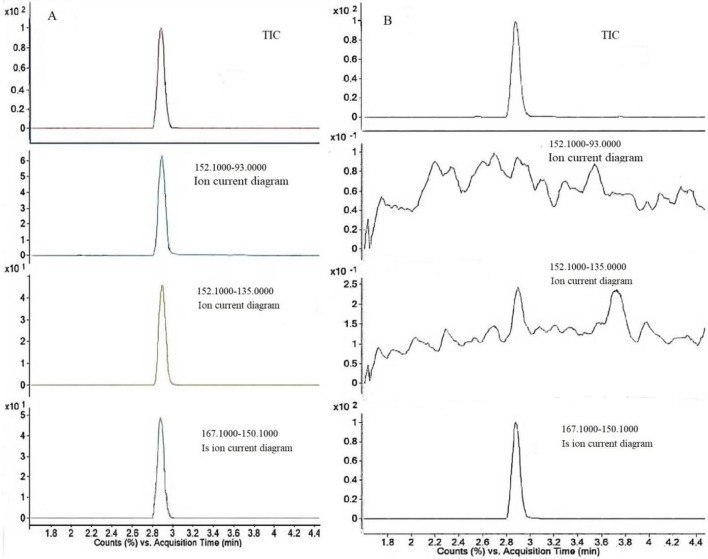
Representative optimized ion chromatograms: **(A)** Blank plasma spiked with amantadine at 5.0 ng/mL (matrix standard) and amantadine-D_15_ (internal standard) at 5.0 ng/mL; **(B)** Blank human plasma spiked with amantadine-D_15_ (internal standard) at 5.0 ng/mL.

The LC-MS/MS system was employed to establish the optimal chromatographic and spectrometric conditions for amantadine by introducing pure standard solutions into the system. Consistent with the common approach for small, basic amine compounds such as amantadine, electrospray ionization (ESI) in positive mode was primarily evaluated. As expected, the positive ionization mode yielded significantly higher sensitivity with minimal background noise compared to the negative mode, which can be attributed to the efficient protonation of amantadine’s amine group. This aligns with the established preference in pharmacokinetic studies, where maximizing sensitivity is paramount for the detection of low-concentration analytes in biological matrices. Initially, precursor ions were detected, followed by the identification of product ions at different collision energies. Among these transitions, the most intense one (152.1→135.1 for amantadine; 167.1→150.1 for amantadine-D_15_) was selected as the quantifier ion to ensure an optimal lower limit of quantification (LLOQ). In accordance with standard bioanalytical guidelines, the peak with the second-highest intensity was designated as the qualifier ion for confirmatory purposes. The multiple reaction monitoring (MRM) transitions and dwell times were then automatically optimized by the instrument based on these parameters to achieve optimal signal-to-noise ratios. The final MRM transitions are summarized in Table 1. Representative chromatograms for spiked plasma (5.0 ng/mL) and blank matrix are presented in Figure 3.

### 3.3 Optimization of sample preparation: Protein precipitation and extraction

Efficient sample preparation is critical to mitigating matrix effects and ensuring assay specificity when analyzing amantadine in biological plasma, which contains abundant proteins and endogenous interferents.

#### 3.3.1 Selection of protein precipitation reagent

**FIGURE 4:**
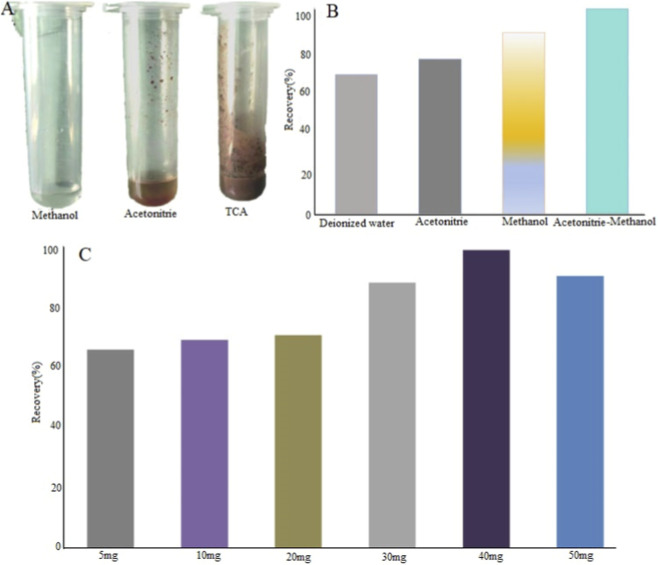
Pre-treatment condition optimization diagram **(A)** Optimization of the precipitant; **(B)** Optimization of extraction reagents; **(C)** Optimizing the amount of adsorbent.

To deproteinize the plasma matrix, three common precipitants - methanol (MeOH), acetonitrile (ACN), and trichloroacetic acid (TCA) - were compared based on the clarity of the resulting supernatant and the compactness of the protein precipitate. As quantitatively demonstrated in Figure 4A, methanol exhibited the highest protein precipitation efficiency, forming a well-defined pellet that facilitated easy and complete transfer of the supernatant. While acetonitrile is widely adopted as a precipitant in bioanalysis, it resulted in a slightly more diffuse pellet under our experimental conditions. Despite being a strong acid precipitant, TCA was excluded due to concerns regarding potential analyte degradation, the introduction of non-volatile salts that could be detrimental to the longevity of the mass spectrometry (MS) system, and the necessity for additional neutralization steps. Therefore, methanol was selected as the optimal precipitant for its superior protein removal, compatibility with subsequent LC-MS/MS analysis, and operational simplicity.

#### 3.3.2 Optimization of analyte extraction solvent

Following protein precipitation, the extraction efficiency of amantadine from the precipitated matrix was optimized, taking into account amantadine’s physicochemical properties-high solubility in organic solvents and relative stability. Four extraction media were assessed: water, pure acetonitrile, pure methanol, and an acetonitrile-methanol mixture (2:1, v/v). The recovery rates, determined by comparing the analyte response from spiked plasma samples processed through the entire PPT protocol versus post-extraction spiked samples, are presented in Figure 4B.

Water showed poor recovery (<50%), consistent with amantadine’s low aqueous solubility. Both pure acetonitrile and pure methanol provided satisfactory recoveries exceeding 80%. Notably, the acetonitrile-methanol mixture (2:1, v/v) achieved a near-quantitative mean recovery of approximately 100%, with significantly reduced relative standard deviation (RSD), indicating higher precision and robustness. This synergistic effect can be attributed to the combined solvent properties: acetonitrile efficaciously penetrates the protein pellet and solubilizes residual analytes, while methanol enhances the solubility and extraction of the target compound. Furthermore, this specific mixture promoted excellent phase separation after centrifugation, yielding a clear supernatant with minimal co-precipitation of interfering lipids or residual proteins, as evidenced by low matrix effect values in subsequent validation. Consequently, the acetonitrile-methanol (2:1, v/v) mixture was adopted as the final extraction solvent, balancing maximal recovery, minimal matrix interference, and procedural efficiency.

### 3.4 Optimization of clean-up strategy: Comparative evaluation of SPE and QuEChERS with a novel ZIF-8/C18 hybrid adsorbent

#### 3.4.1 Rationale for clean-up strategy and adsorbent design

Plasma is a highly complex matrix containing proteins, lipids, salts, and numerous endogenous metabolites that can cause severe ion suppression/enhancement (matrix effects) in LC-MS/MS analysis, thereby compromising assay accuracy and reproducibility. An efficient cleanup procedure is therefore paramount. This study comparatively assessed two principal strategies: conventional solid-phase extraction (SPE) and a modified QuEChERS (Quick, Easy, Cheap, Efficacious, Rugged, and Safe) approach. The QuEChERS protocol was enhanced by employing a hybrid adsorbent consisting of conventional C18 and synthetic zeolitic imidazolate framework-8 (ZIF-8).

C18, a reversed-phase silica-based sorbent, is efficacious for removing non-polar interferents like lipids and sterols. However, its selectivity and capacity for diverse polar matrix components can be limited. To address this, we incorporated ZIF-8, a subclass of metal-organic frameworks (MOFs). ZIF-8 features a sodalite zeolite topology, an exceptionally high surface area (∼1300–1800 m^2^/g), and a hydrophobic microporous structure. These properties endow the material with an outstanding adsorption capacity towards a wide array of interfering compounds. This is achieved through multiple mechanisms, encompassing size-exclusion, π-π interactions, and hydrophobic effects. Consequently, it emerges as a highly promising complementary adsorbent for the clean-up of complex biological samples.

#### 3.4.2 Optimization of hybrid adsorbent composition

The dosage of the hybrid adsorbent was systematically optimized. A fixed amount of 10 mg C18 per 1 mL of plasma was used based on established protocols. The amount of ZIF-8 was then varied (5, 10, 20, 30, 40, and 50 mg) while processing blank plasma samples spiked with 5 ng/mL amantadine. Extraction recovery served as the primary optimization criterion.

As presented in Figure 4C, the recovery of amantadine increased significantly with the ZIF-8 amount, reaching a plateau of >90% at 40 mg. A further increase to 50 mg resulted in a notable decrease in recovery. This parabolic trend can be attributed to the adsorption dynamics of ZIF-8: at the optimal dosage (40 mg), the abundant active sites were sufficiently occupied by matrix interferents, allowing the target analyte to remain in solution. An excessive quantity of adsorbent (50 mg) likely led to non-specific adsorption of amantadine molecules onto the remaining unoccupied active sites, subsequently reducing the recovery rate. Therefore, a hybrid adsorbent formulation consisting of 10 mg of C18 and 40 mg of ZIF-8 per 1 mL of plasma was determined to be optimal for the QuEChERS cleanup procedure.

#### 3.4.3 Comparative performance of SPE and optimized QuEChERS

The performance of the optimized QuEChERS method was directly compared against a conventional SPE protocol (e.g., using Oasis HLB cartridges). The results of this comparative evaluation are summarized in Table 2.

**TABLE 2:** Comparative evaluation of SPE and Optimized QuEChERS.

Evaluation parameter	Solid-phase extraction (SPE)	QuEChERS with ZIF-8/C18	Comparative implication
Mean extraction recovery	101.7%	∼90%	SPE offers excellent, near-quantitative recovery. QuEChERS recovery is well within the accepted bioanalytical range (85%–115%)
Clean-up efficiency	High, yielding clean extracts	High, efficaciously removes diverse interferents	Both provide sufficient clean-up. ZIF-8’s broad-spectrum adsorption complements C18’s selectivity
Matrix effect (ion suppression)	Minimized due to selective elution	Efficaciously controlled, as evidenced by low signal variation	Both strategies adequately mitigate critical LC-MS/MS matrix effects
Operational process	Multi-step (condition, load, wash, elute, dry, reconstitute), prone to column clogging with complex plasma	Simple: Adsorbent addition, vortex, centrifuge, supernatant collection	QuEChERS is inherently simpler, faster, and more robust against sample particulates
Cost & throughput	Higher cost per sample; moderate throughput due to manual steps	Lower cost; highly amenable to batch processing and automation	QuEChERS presents clear advantages for large-scale or routine analysis
Environmental impact	Consumes more organic solvents and plastic ware	Aligns with green analytical chemistry principles (less solvent, minimal waste)	QuEChERS is the more sustainable choice

#### 3.4.4 Justification for method selection

Based on the comprehensive comparison, the optimized QuEChERS method was selected for the final assay. While SPE yielded marginally higher recovery, its operational complexity, higher cost, and vulnerability to column clogging present practical limitations for high-throughput applications. The QuEChERS protocol, enhanced with the ZIF-8/C18 adsorbent, achieved a more than satisfactory recovery (∼90%) with excellent clean-up efficiency. Its paramount advantages lie in procedural simplicity, speed, cost-effectiveness, and superior scalability for batch processing, making it the more pragmatic and sustainable choice for the determination of amantadine in plasma without compromising on data quality.

### 3.5 Assessment and mitigation of matrix effects

#### 3.5.1 Significance and evaluation principle

Matrix effect (ME) represents a pivotal challenge in quantitative bioanalysis via LC-MS/MS, primarily arising from the co-elution of endogenous or exogenous matrix components with the target analyte, which can alter its ionization efficiency in the electrospray source (Zhu et al., 2025; Ververi et al., 2024). This phenomenon, manifesting as ion suppression or enhancement, directly compromises the accuracy, precision, and reproducibility of analytical measurements. Therefore, systematic evaluation of ME is an indispensable component of bioanalytical method validation.

In this study, the magnitude of the matrix effect was quantitatively assessed using the post-extraction spike method, which is recommended by international guidelines (e.g., FDA, EMA) for its reliability. The Matrix Effect assessment is based on comparing the analyte response in a pre-processed (extracted) blank matrix sample that is subsequently spiked with the analyte, to the response of an equivalent analyte concentration in a pure solvent solution. The Matrix Effect (%) is determined as Equation 1.

$$\text{ME }\left(\%\right)=\left(\text{ Peak Response of Post}‐\text{Extraction Spiked Sample }/\times \text{ Peak Response of Neat Solvent Standard }\right)\times 100\%$$ (1)

According to established bioanalytical criteria, an ME value between 80% and 120% (or 0.8–1.2) is generally considered acceptable, indicating negligible or mild matrix interference (Ding et al., 2025). Values below 80% signify ion suppression, while values above 120% indicate ion enhancement.

#### 3.5.2 Results and interpretation for amantadine

The matrix effect was assessed across multiple lots of blank human plasma (n ≥ 6) at low and high concentration levels of amantadine. The determined ME value for amantadine was 92% (or 0.92 on a ratio scale), with a low relative standard deviation (RSD <15%).

This result falls well within the acceptable range of 80%–120%, demonstrating that the optimized sample preparation protocol-specifically, the QuEChERS clean-up with the ZIF-8/C18 hybrid adsorbent developed in Section 3.4.2-efficaciously removed the vast majority of ionizable matrix interferents. The negligible matrix effect observed validates the efficiency of our clean-up strategy and confirms that the ionization process of amantadine is not adversely impacted by co-eluting plasma components under the finalized chromatographic conditions.

#### 3.5.3 Contextual discussion and method robustness

A matrix effect (ME) of 92%, which can be considered negligible, strongly attests to the robustness of the method we have developed. This implies that the quantitative results obtained are not distorted by the unpredictable variations in plasma composition that can occur across different individuals or sample batches-a common issue that poses challenges in clinical and pharmacokinetic studies.

While some reported methods for amantadine achieve adequate sensitivity with simpler protein precipitation, they often report more pronounced matrix effects requiring compensation via extensive use of stable isotope-labeled internal standards (SIL-IS). The effectiveness of our clean-up step in mitigating ME reduces the method’s reliance on perfect isotopologue correction alone and enhances its overall reliability.

It is vital to note that the evaluation was performed using amantadine-D_15_ as the internal standard, which, due to its nearly identical chemical and chromatographic behaviors, efficaciously corrects for any residual minor matrix effects or instrument variability, thereby further ensuring quantitative accuracy.

### 3.6 Stability studies

The stability of analytes in biological matrices is a pivotal pre-analytical factor that can significantly impact the reliability of pharmacokinetic and bioanalytical data. Our investigation into the stability of amantadine in human plasma under various storage conditions revealed a degradation profile that is dependent on both temperature and concentration, emphasizing the significance of implementing stringent sample handling protocols (Table 3).

**TABLE 3:** Stability testing results of amantadine in plasma (n = 3).

Type	Time	5 ng/mL		50 ng/mL		500 ng/mL	
		Accuracy/%	RSD/%	Accuracy/%	RSD/%	Accuracy/%	RSD/%
Room temperature stability (20 °C)/h	12	90.2	4.3	91.5	3.4	95.6	2.3
	24	87.9	5.4	92.3	1.2	94.3	3.7
	48	85.6	7.2	89.6	2.8	92.9	1.8
Refrigerated stability (2 °C–8 °C)/d	1	91.4	5.6	93.5	3.8	94.2	4.6
	2	92.0	6.1	92.8	4.5	95.1	3.9
	5	90.9	7.0	94.0	3.2	93.6	5.8
	7	89.8	4.8	91.5	5.9	92.8	4.1
Freeze-thaw stability (−20 °C)/cycle	1	91.5	3.2	93.5	6.0	94.8	3.9
	2	90.2	4.8	92.8	5.7	95.1	4.2
	3	91.8	4.1	94.0	5.8	93.2	3.1

At room temperature (20 °C), amantadine demonstrated limited stability. A noticeable decrease in concentration was observed after 12 h, progressing further at 24 and 48 h. This instability was more pronounced at the lower concentration of 5 ng/mL, suggesting that degradation processes may have a more significant relative impact on samples near the lower limit of quantification (LLOQ). Higher concentrations exhibited better stability, likely due to a lower proportional loss from any degradation mechanism. This concentration-dependent stability highlights a particular risk for the accurate measurement of samples with low drug levels, which are often encountered in terminal phase pharmacokinetic studies.

Improved stability was observed under refrigerated conditions (2 °C–8 °C) and following freeze-thaw cycles. While some reduction in measured concentration occurred under these conditions, the degree of change was markedly less compared to ambient storage, indicating that low temperatures efficaciously slow the degradation kinetics. The relative stability under refrigerated and frozen conditions is consistent with the general practice of cold-chain storage for plasma samples. Nevertheless, the fact that a measurable decrease was still detected, even under such precisely controlled conditions, is noteworthy. This gradual and persistent decline may potentially be ascribed to a combination of factors, including slow-paced chemical degradation processes, residual enzymatic activities not entirely suppressed by the anticoagulant, or the adsorption of analytes onto the inner surfaces of the containers.

The findings have direct implications for bioanalytical practice. Although storage at 2 °C–8 °C or −20 °C/-80 °C is indispensable for sample preservation, it does not guarantee indefinite stability. The data at hand indicate that, whenever feasible, prolonged storage should be avoided, even when samples are kept under refrigerated conditions. This minimizes the time window for pre-analytical degradation and reduces the risk of obtaining artificially low concentrations that do not reflect the *in vivo* state at the time of blood draw. This kind of inaccuracies could lead to erroneous conclusions regarding pharmacokinetic parameters, such as half-life or clearance, and compromise the validity of bioequivalence or therapeutic drug monitoring investigations.

In conclusion, amantadine in plasma is susceptible to time- and temperature-dependent degradation. To ensure analytical integrity, a robust standard operating procedure (SOP) should mandate immediate plasma separation after blood collection, followed by prompt analysis or rapid transfer to frozen storage at ≤ −20 °C. For long-term studies, establishing institution-specific stability data for the entire storage period, including freeze-thaw cycles, is essential to define validated storage timelines.

### 3.7 Validation of method effectiveness

A robust and reliable method for the quantification of amantadine in human plasma has undergone a thorough validation process in strict accordance with established standard criteria. This validation has unequivocally demonstrated that the method possesses performance characteristics that render it highly suitable for application in both clinical and pharmacokinetic research settings.

The method exhibits excellent linearity across a clinically pertinent concentration range of 0.5–20 ng/mL, as evidenced by a correlation coefficient (R^2^) of 0.9978. This wide dynamic range, coupled with a lower limit of quantification (LOQ) of 0.5 ng/mL, ensures the method’s capability to accurately measure amantadine concentrations from the terminal elimination phase up to peak plasma levels following therapeutic dosing. The established dilution procedure for samples exceeding the upper limit of quantification (ULOQ) ensures the accuracy of measurements for high-concentration samples without the need for re-analysis, enhancing workflow efficiency.

The precision and accuracy of the method, ascertained via spike-recovery tests, conform to internationally recognized bioanalytical guidelines (Table 4). The recovery rates, which span from 94.5% to 110.1% across four distinct concentration levels, serve as a strong indicator of minimal matrix interference and high method accuracy. More importantly, the reproducibility of the method is confirmed by the low variability observed both within a single analytical run (intra-day RSD: 2.8%–5.8%) and between different runs conducted over several weeks (inter-day RSD: 5.4%–7.8%). These RSD values, which are well within the typical acceptance criterion of ≤15% (and ≤20% at the LOQ), firmly underscore the method’s robustness and long-term reliability. It can be inferred that the successful utilization of an internal standard plays a pivotal role in achieving this high level of precision, as it effectively corrects for potential variations in sample preparation and instrument response.

**TABLE 4:** Recovery and repeatability of amantadine at different additive concentrations (n = 7).

Spiked (ng/mL)	The first determination			The second determination			RSD (%)
	Average measured value (ng/mL)	Average recovery Rate/%	RSD/%	Average measured val (ng/mL)	Average recovery Rate/%	RSD/%	
0.5	0.49	97.9	2.7	0.47	94.1	3.1	7.8
1.0	0.94	93.9	3.0	0.96	95.9	2.8	9.6
2.0	1.95	97.5	4.5	1.98	99.0	5.8	6.5
5.0	5.06	101.1	3.3	4.96	99.2	4.3	5.4

**FIGURE 5:**
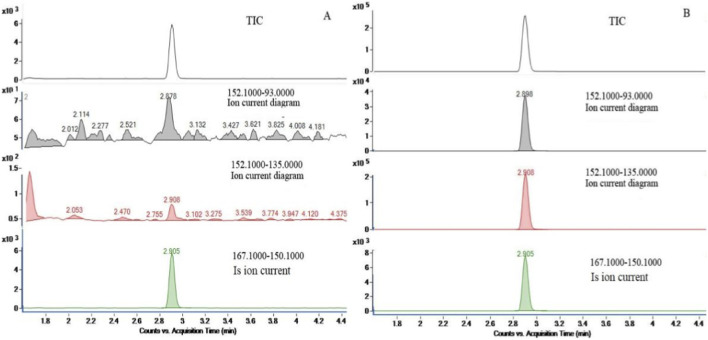
Representative chromatogram: **(A)** LOD 0.15 ng/mL; **(B)** ULOQ 20.0 ng/mL.

The combination of a low LOD (0.15 ng/mL), adequate sensitivity (LOQ of 0.5 ng/mL), wide linear range, and satisfactory accuracy and precision establishes this method as a fit-for-purpose tool for the quantitative analysis of amantadine in plasma (Figure 5). Its performance parameters are fully adequate to support critical applications such as therapeutic drug monitoring, bioavailability/bioequivalence studies, and detailed pharmacokinetic investigations. Future work may involve applying this validated method to such real-world sample analyses and potentially extending its validation to include other biological matrices.

### 3.8 Pharmacokinetic study and mathematical modeling

**FIGURE 6:**
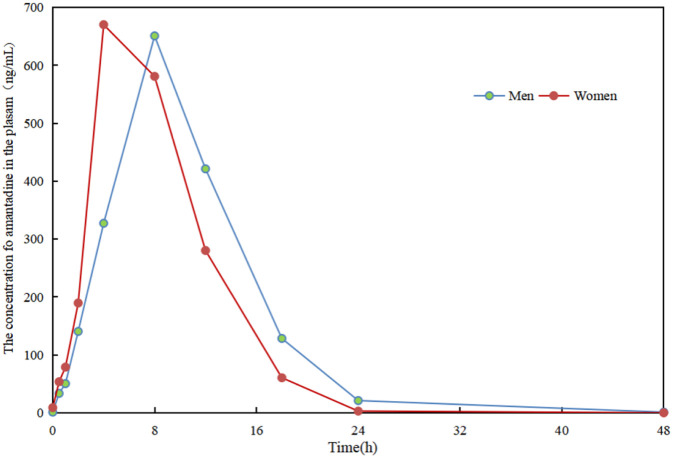
Changes in plasma amantadine concentrations over sampling time.

The time-dependent plasma concentrations of amantadine in male and female subjects are presented in Figure 6. Following oral administration, plasma concentrations exhibited a characteristic profile, increasing to a maximum (C_max_) before declining in a monophasic manner, which is indicative of rapid absorption followed by elimination.

Pharmacokinetic analysis revealed that the concentration-time data were best described by a first-order kinetic model for both absorption and elimination phases, as confirmed by high correlation coefficients (R^2^ > 0.9) for the fitted equations (Table 5). Key derived parameters, including the geometric means of time to reach peak concentration (T_max_), elimination half-life (T_1/2_), and clearance (CL), followed consistent trends between genders.

**TABLE 5:** Fitting equations of amantadine absorption and elimination kinetics.

Gender	Absorption equations	Correlation coefficient *(R* ^2^)	Degradation equations	Correlation coefficient *(R* ^2^)
M	y=84.304x−20.80	0.9981	$c=2130.8e^{-0.161t}$	0.9722
F	y=166.38x−49.26	0.9423	$c=2083.1e^{-0.149t}$	0.9321

Notably, a gender difference was observed in the absorption profile. Female subjects achieved a higher peak concentration (C_max_ = 670.23 ng/mL) at an earlier T_max_ (4 h), whereas male subjects reached a slightly lower C_max_ (650.87 ng/mL) at a later T_max_ (8 h). Despite this difference in T_max_, the overall elimination kinetics were similar between groups. The rapid absorption observed, particularly in females, supports a prompt onset of pharmacological action. Moreover, the substantial decline in plasma concentrations by 24 h post-administration suggests efficient clearance, which may correlate with a reduced risk of accumulation and related side effects during once- or twice-daily dosing regimens.

The well-established first-order kinetic model provides a robust framework for predicting real-time plasma concentrations of amantadine in treated patients. This predictive capability offers valuable clinical utility, potentially aiding in the optimization of dosing strategies to enhance therapeutic efficacy while minimizing adverse effects.

### 3.9 Application of the mathematical model

The principal aim of constructing a pharmacokinetic model is to facilitate the real-time monitoring of amantadine therapy, thereby enabling more precise and individualized patient management. To substantiate the practical utility of the established first-order kinetic model, a clinical assessment was carried out on eight influenza patients who received a standard oral dose of 200 mg (2 × 100 mg tablets).

Plasma concentrations of amantadine were measured at 2, 8, and 24 h post-administration using the validated bioanalytical method described previously. These experimentally determined concentrations were juxtaposed with the values predicted by the mathematical model. Predictions were generated by applying the absorption-phase equations to the 2-h and 8-h time points, and the elimination-phase equation to the 24-h time point, with the initial measurement serving as a baseline.

The comparative results, as summarized in Table 6, offer a profound critical insight into the relationship between population-based models and individual pharmacokinetics. Although the model generated consistent predicted concentrations across all patients at each nominal time point, which is a reflection of its foundation on average population parameters, the actual measured concentrations demonstrated substantial inter-individual variability. This observed dispersion is attributable to well-known physiological and pathophysiological factors that influence drug disposition, such as variations in gastric emptying, hepatic enzyme activity, renal function, and body composition.

**TABLE 6:** Concentration of amantadine in plasma of patients at different time points after administration.

NO.	Age	Gender	Cmeasuredt = 2 h (ng/mL)	Cmeasuredt = 8 h (ng/mL)	Cmeasuredt = 24 h (ng/mL)	Cpredictedt = 2 h (ng/mL)	Cpredictedt = 8 h (ng/mL)	Cpredictedt = 24 h (ng/mL)
1	25	M	138.9	662.9	38.9	147.81	653.6	44.7
2	36	M	138.3	658.9	43.2			
3	46	M	136.5	636.7	43.1			
4	58	M	130.5	600.3	50.1			
5	22	F	275.3	632.9	49.2	283.5	632.4	58.30
6	37	F	281.5	658.1	50.9			
7	45	F	258.9	628.9	60.3			
8	60	F	243.8	635.4	57.8			

Despite this inherent individual variability, the model successfully captured the central trend of amantadine’s pharmacokinetic profile, accurately depicting the sequence of rapid absorption followed by progressive elimination. This capability confirms the model’s robustness for describing typical kinetic behavior. Consequently, it serves as a valuable clinical tool, providing physicians with a rational, quantitative framework to support therapeutic decision-making. By comparing observed patient drug levels against the model’s predicted trajectory, clinicians can better assess the adequacy of exposure, identify potential outliers in drug handling (e.g., poor absorbers or rapid metabolizers), and make more informed adjustments to dosing regimens to optimize efficacy and minimize toxicity, moving towards a more personalized treatment approach.

## 4 Conclusion

In this study, a highly sensitive and reliable LC-MS/MS method was successfully developed and validated for the quantification of amantadine in human plasma. An efficient sample preparation strategy was adopted by means of acetonitrile and methanol (3:1, v/v) as the extraction solvent and incorporated a novel metal-organic framework adsorbent, ZIF-8, based on the QuEChERS principle. The unique nanoporous architecture, large pore size, and high specific surface area of ZIF-8 efficaciously adsorb plasma matrix interferences, thereby enhancing purification efficiency. This approach, combined with internal standard calibration, results in a streamlined workflow, excellent reproducibility, and performance metrics that fully comply with the rigorous standards required for clinical bioanalysis.

This rigorously validated analytical method has been effectively employed to monitor amantadine concentrations in pharmacokinetic investigations. In healthy volunteers, amantadine exhibited characteristic biphasic pharmacokinetic behavior, comprising an initial rapid absorption phase (0–8 h) followed by a first-order elimination phase (8–48 h). A mathematical model that seamlessly integrated these two distinct phases was constructed and subsequently validated. This model has proven its worth by enabling the real-time prediction of plasma amantadine levels. This predictive capability provides clinicians with a practical tool to better understand individual drug exposure, thereby supporting the optimization of dosing regimens and potentially improving therapeutic efficacy while minimizing adverse effects.

Nevertheless, the present study is not devoid of limitations. The pharmacokinetic data and model validation were derived exclusively from a cohort of healthy volunteers aged 18–60 years. Thus, the findings may not be directly generalizable to key patient populations such as pediatric or elderly individuals, or those with specific comorbidities (e.g., renal or hepatic impairment) that could significantly alter drug disposition. Future research endeavors ought to concentrate on assessing the pharmacokinetic variability of amantadine in these diverse populations. Furthermore, research into the influence of genetic polymorphisms (e.g., in metabolizing enzymes or transporters) and other physiological factors on its absorption and elimination is warranted to enable truly personalized dosing strategies.

## Data Availability

The original contributions presented in the study are included in the article/supplementary material, further inquiries can be directed to the corresponding author.
